# Greatly reduced risk of EBV reactivation in rituximab-experienced recipients of alemtuzumab-conditioned allogeneic HSCT

**DOI:** 10.1038/bmt.2016.19

**Published:** 2016-02-22

**Authors:** D M Burns, S Rana, E Martin, S Nagra, J Ward, H Osman, A I Bell, P Moss, N H Russell, C F Craddock, C P Fox, S Chaganti

**Affiliations:** 1 Institute of Cancer and Genomic Sciences, University of Birmingham, Birmingham, UK; 2 Centre for Clinical Haematology, University Hospitals Birmingham NHS Trust, Birmingham, UK; 3 Cancer Research UK Clinical Trials Unit, University of Birmingham, Birmingham, UK; 4 Health Protection Agency Laboratory, Birmingham Heartlands Hospital, Birmingham, UK; 5 Institute of Immunology and Immunotherapy, University of Birmingham, Birmingham, UK; 6 Centre for Clinical Haematology, Nottingham University Hospitals NHS Trust, Nottingham, UK

## Abstract

EBV-associated post-transplant lymphoproliferative disease (PTLD) remains an important complication of allogeneic haematopoietic stem cell transplantation (allo-HSCT). We retrospectively analysed the incidence and risk factors for EBV reactivation in 186 adult patients undergoing consecutive allo-HSCT with alemtuzumab T-cell depletion at a single centre. The cumulative incidence of EBV reactivation was 48% (confidence interval (CI) 41–55%) by 1 year, with an incidence of high-level EBV reactivation of 18% (CI 13–24%); 8 patients were concurrently diagnosed with PTLD. Amongst patients with high-level reactivation 31/38 (82%) developed this within only 2 weeks of first EBV qPCR positivity. In univariate analysis age⩾50 years was associated with significantly increased risk of EBV reactivation (hazard ratio (HR) 1.54, CI 1.02–2.31; *P*=0.039). Furthermore, a diagnosis of non-Hodgkin lymphoma (NHL) was associated with greatly reduced risk of reactivation (HR 0.10, CI 0.03–0.33; *P*=0.0001) and this was confirmed in multivariate testing. Importantly, rituximab therapy within 6 months prior to allo-HSCT was also highly predictive for lack of EBV reactivation (HR 0.18, CI 0.07–0.48; *P*=0.001) although confounding with NHL was apparent. Our data emphasise the risk of PTLD associated with alemtuzumab. Furthermore, we report the clinically important observation that rituximab, administered in the peri-transplant period, may provide effective prophylaxis for PTLD.

## Introduction

Post-transplant lymphoproliferative disease (PTLD) remains a life-threatening complication of allogeneic haematopoietic stem cell transplant (allo-HSCT).^1, 2, 3^ In this setting, almost all cases arise from donor-derived B cells, transformed by the ubiquitous double-stranded DNA gamma-herpesvirus EBV. In healthy individuals, EBV establishes a lifelong latent infection within resting memory B cells and is controlled by potent virus-specific T-cell responses. However, in the state of T-cell immunocompromise that follows allo-HSCT, latently infected B cells may exhibit opportunistic expansion. Viral reactivation may be detected as an increase in the level of circulating viral genomes, EBV DNAemia, as measured by EBV-specific quantitative PCR (EBV qPCR). Established PTLD has conventionally been associated with high rates of mortality. The incidence of PTLD arising after allo-HSCT varies significantly in relation to graft T-cell depletion (TCD), ranging from less than 1% without TCD to more than 10% with TCD in some series.^4, 5, 6, 7, 8, 9, 10, 11, 12, 13, 14, 15, 16^ Other important risk factors include the use of unrelated and/or HLA-mismatched donors, the occurrence of severe GvHD and older age.

Of the different methods of TCD in current use, anti-thymocyte globulin has been associated with substantially elevated rates of EBV reactivation and PTLD.^4, 12, 14, 16^ Meanwhile, the anti-CD52 monoclonal Ab Campath is thought to confer a lower incidence of PTLD, reportedly 0.4–1.3% in initial studies.^4, 5^ Unlike anti-thymocyte globulin, which selectively depletes T cells, Campath targets all lymphocytes. Therefore, the risk of disease associated with Campath may be mitigated by simultaneous depletion of B cells. However, initial studies examining the risks associated with Campath involved first and second generation rat antibodies (Campath-1M and -1G). Meanwhile, relatively few studies have examined the third generation humanised Ab Campath-1H (alemtuzumab), which has superseded the other Abs in routine clinical practice.^17^ Alemtuzumab has a much longer half-life than Campath-1G (15–21 days, compared with less than 24 h) and causes prolonged delays in the reconstitution of both general^18, 19^ and EBV-specific T-cell immunity.^20^ Consequently, it might contribute to an increased risk of PTLD compared with earlier antibodies.

Introduction of the anti-CD20 monoclonal Ab rituximab has significantly improved PTLD-related mortality.^21^ In particular, administration of rituximab to individuals who develop EBV reactivation, pre-empting the development of PTLD, has led to a marked reduction in mortality relative to historic cohorts.^11, 22, 23, 24, 25^ However, there remains marked inter-centre variation in EBV qPCR methodology and a lack of consensus as to the optimal virus load thresholds to trigger pre-emptive intervention.^26, 27, 28^ An alternative strategy for the prevention of PTLD is prophylactic infusion of donor-derived EBV-specific cytotoxic T lymphocytes prepared *in vitro*.^29^ Although efficacious, this approach has not been widely adopted, largely because of the logistical and financial burdens involved. It has also been proposed that peri-transplant rituximab (delivered shortly before or after transplant) might be an effective method of prophylaxis.^30, 31^

To explore these issues, work was undertaken to define incidence, kinetics and risk factors for EBV reactivation and PTLD amongst adult patients undergoing alemtuzumab TCD allo-HSCT at a major UK transplant centre.

## Subjects and methods

This retrospective study examined adult patients undergoing consecutive TCD allo-HSCT at University Hospital Birmingham between May 2009 and September 2012. The study was registered with University Hospital Birmingham Research and Development department, and participants provided written consent for clinical data collection. All patients received myeloablative or reduced-intensity conditioning according to institutional protocols, followed by PBSC graft infusion. TCD was carried out with *in vivo* alemtuzumab, comprising 10 mg daily intravenously from day -7 to day -3 before stem cell infusion. GvHD prophylaxis comprised cyclosporine, with or without methotrexate, or mycophenolate mofetil. All patients received aciclovir prophylaxis for a minimum of 3 months following transplant.

Patients were monitored using EBV qPCR whole blood assay of the EBV polymerase gene BALF5.^32^ EBV qPCR testing was scheduled every 1–2 weeks for the first 6 months post transplant and intermittently thereafter. EBV reactivation was defined as a single positive EBV qPCR result exceeding the assay limit of sensitivity of 500 genomes/mL. High-level EBV reactivation was defined as a single result ⩾20 000 genomes/mL—a locally determined threshold selected on the basis of prior experience of the assay performed in this clinical setting. Patients exceeding the high-level EBV threshold were assessed for possible PTLD and received pre-emptive treatment with up to 4 weekly infusions of rituximab 375 mg/m^2^. Cases of PTLD were diagnosed in accordance with published definitions for biopsy-proven or probable disease arising after allo-HSCT, the latter including radiologic evidence of disease in association with EBV DNAemia.^33^

Cumulative incidence of EBV reactivation was estimated taking the competing risk of death into account. Cumulative incidence curves were compared using the Log Rank test. Univariate and multivariate analysis of risk factors for EBV reactivation were performed using Cox proportional hazards modelling. Acute GvHD was treated as a time-dependent covariate. The effects of pre-transplant or post-transplant (pre-emptive) rituximab therapy on overall survival or non-relapse mortality was analysed using Cox testing, considering post transplant rituximab as a time-dependent covariate. All analyses were carried out in Stata 12.

## Results

### Incidence and kinetics of EBV reactivation and PTLD

**Figure 1:**
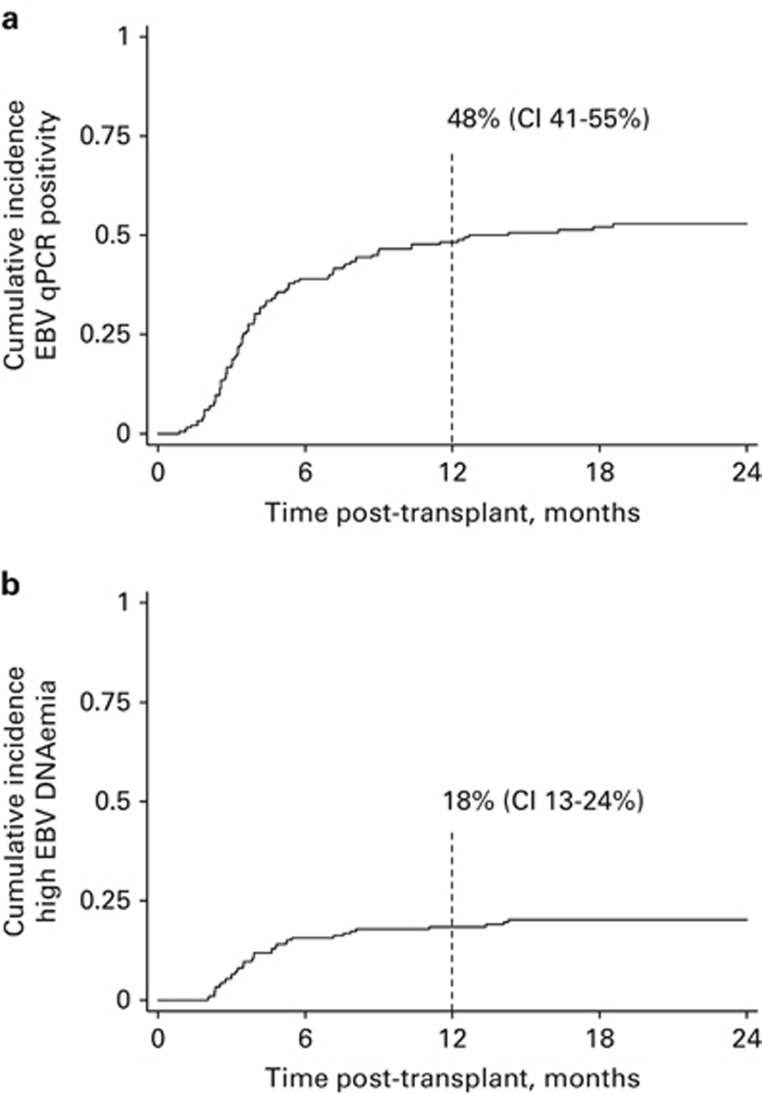
Incidence of EBV reactivation after alemtuzumab TCD Allo-HSCT. The cumulative incidence of EBV DNAemia following allo-HSCT was calculated taking the competing risk of death into account. The plots display incidence for 186 patients who received TCD with alemtuzumab. (**a**) Incidence of EBV qPCR positivity, defined as a single result ⩾500 genomes/mL. (**b**) Incidence of high-level EBV DNAemia, defined as a single result ⩾20 000 genomes/mL.

This study includes 186 adult patients undergoing first allo-HSCT with alemtuzumab TCD at University Hospital Birmingham, UK. Median follow-up was 28 months. Overall survival was 72% at 1 year post transplant, with non-relapse mortality of 2.9% at 100 days and 11.5% at 1 year. Patient characteristics are summarised in Table 1. Monitoring revealed a cumulative incidence of EBV reactivation (⩾500 genomes/mL) of 48% (95% confidence interval (CI) 41–55%) at 1 year (Figure 1a). Furthermore, the cumulative incidence of high-level EBV reactivation (⩾20 000 genomes/mL) was 18% (CI 13–24% Figure 1b). In total, 38 patients developed high-level EBV reactivation. Of these, eight were concurrently diagnosed with PTLD (Table 2). The median interval between first EBV load ⩾20 000 copies/mL and radiographically documented disease (comprising computed tomography and/or positron emission tomography–computed tomography imaging in all cases) was only 7 days (range 1–16 days). Five (63%) patients had B symptoms documented at presentation and 7/8 (88%) had stage ⩾3 disease.

**Figure 2:**
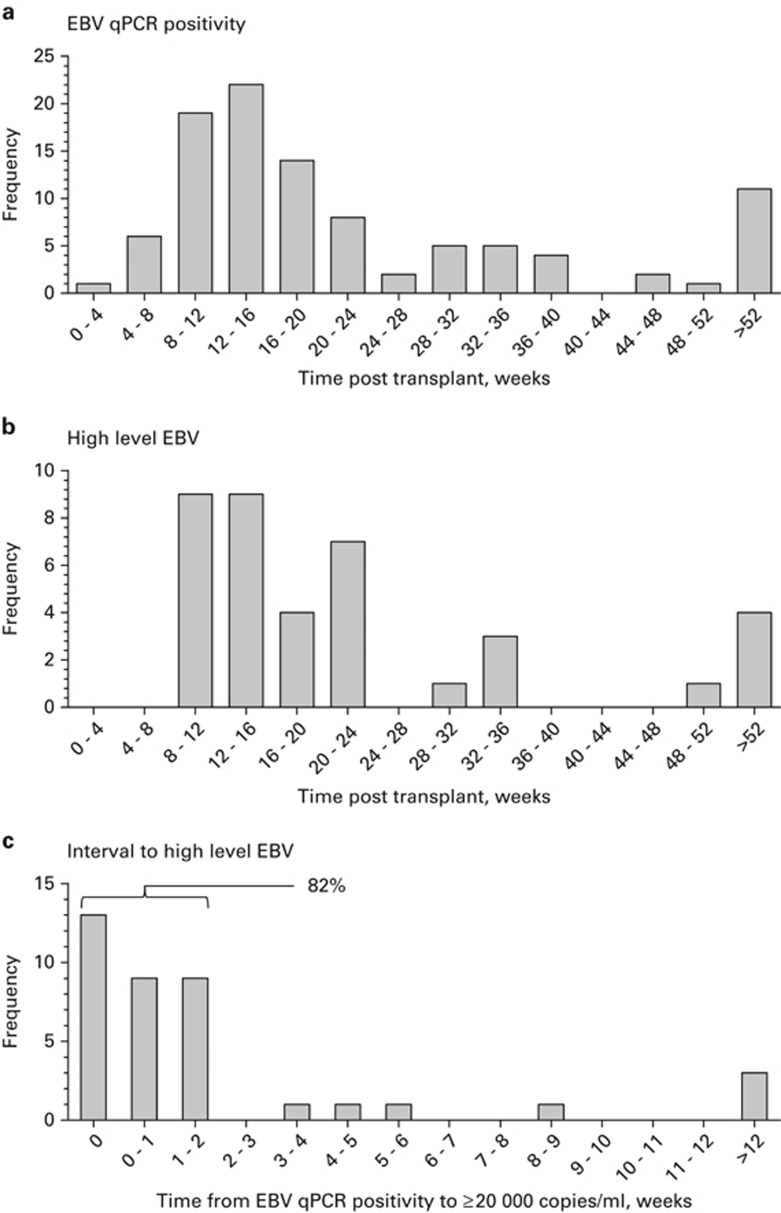
Kinetics of EBV reactivation after allo-HSCT. (**a**) Interval from transplant to first EBV qPCR-positive test, ⩾500 genomes/mL. (**b**) Interval from transplant to high-level EBV DNAemia, ⩾20 000 copies/mL. (**c**) Interval from first EBV qPCR-positive test to high-level EBV DNAemia. The proportion of patients exhibiting high-level EBV DNAemia within 2 weeks of initial EBV qPCR positivity is indicated.

The kinetics of post-transplant EBV reactivation are summarised in Figure 2. Most reactivations occurred between 8 and 16 weeks after transplant. No cases of high-level EBV reactivation occurred in the first 8 weeks. However, late high-level reactivations, occurring more than a year after transplant, were still observed in 4/38 (11%) patients. Importantly, analysis of the time interval from initial EBV qPCR positivity to high-level EBV reactivation (Figure 2c) revealed that 13/38 (34%) patients already exhibited high-level reactivation at first EBV qPCR positivity, and a further 18 patients developed high-level reactivation within 2 weeks of this. As such, a total of 31/38 (82%) patients developed high-level EBV reactivation within only 2 weeks of first EBV qPCR positivity.

### Pre-emptive management

**Figure 3:**
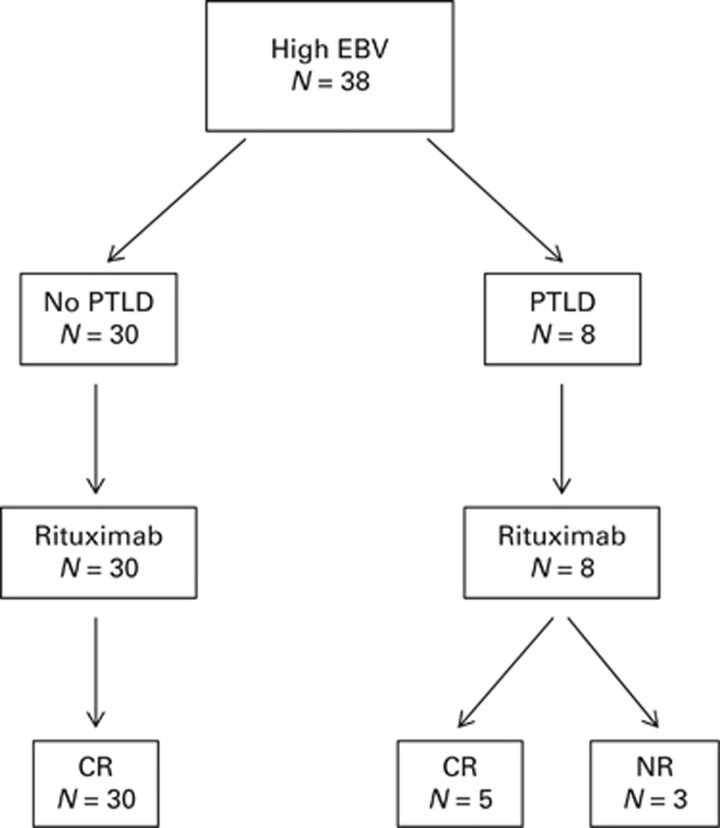
Pre-emptive management of EBV reactivation after allo-HSCT. All patients with EBV DNAemia ⩾20 000 copies/mL were treated with up to 4 weekly infusions of rituximab 375 mg/m^2^. The flowchart summarises outcomes for all treated patients, eight of whom were concurrently diagnosed with PTLD at initiation of therapy. Response is defined as complete and sustained resolution of EBV DNAemia and disease where evident.

All 38 patients with high-level EBV reactivation, including 8 diagnosed with PTLD, were treated pre-emptively with up to 4 weekly infusions of rituximab (Figure 3). Of these, all 30 without evidence of PTLD, and 5/8 (63%) with PTLD, responded—defined as sustained resolution of EBV loads to undetectable levels and complete radiological remission of disease where present. Three patients with PTLD showed refractoriness to rituximab, all of whom died from progressive disease despite further treatment including CHOP chemotherapy in two cases (Supplementary Figure S1).

As some authors have raised concerns about the safety of rituximab following allo-HSCT,^34, 35^ the impact of pre-emptive rituximab on survival was analysed; three patients who died from PTLD were excluded from this analysis to eliminate the effect of PTLD-related mortality. This revealed no significant deleterious effect from pre-emptive rituximab on overall survival (hazard ratio (HR) 1.41, CI 0.79–2.51; *P*=0.239) or non-relapse mortality (HR 1.57, CI 0.62–3.94; *P*=0.338).

### Factors predicting for EBV reactivation

**Figure 4:**
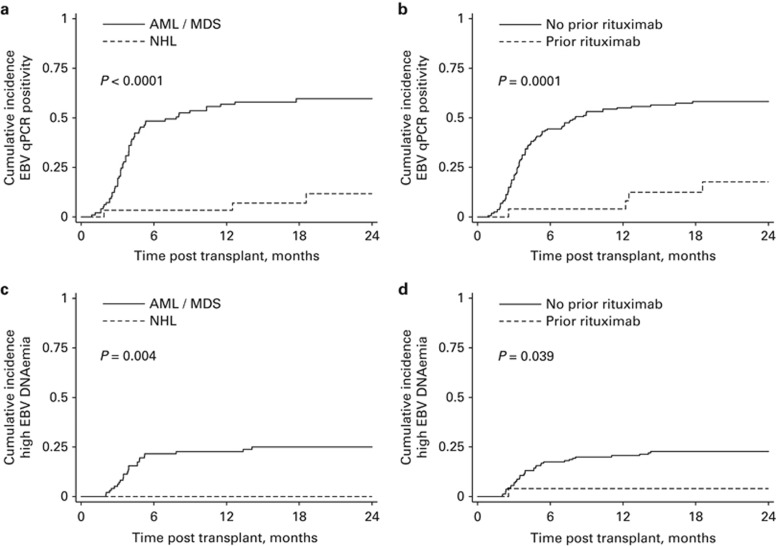
Incidence of EBV reactivation by diagnosis and prior rituximab exposure. Plots show the cumulative incidence of: (**a**) EBV qPCR positivity (⩾500 genomes/mL) in patients with AML/MDS versus NHL, (**b**) EBV qPCR positivity in patients according to rituximab exposure within 6 months prior to transplant, (**c**) high-level EBV DNAemia (⩾20 000 copies/mL) in patients with AML/MDS versus NHL and (**d**) high-level EBV DNAemia according to rituximab exposure within 6 months prior to transplant. Cumulative incidence curves are compared with the Log Rank test.

Baseline predictors of EBV reactivation were analysed using univariate Cox regression (Table 3 and Figure 4). This revealed patients aged ⩾50 years to be at significantly increased risk (HR 1.54, CI 1.02–2.31; *P*=0.039) of viral reactivation. Furthermore, although the intensity of transplant conditioning was not significantly predictive overall, Flu Mel conditioning was associated with increased risk relative to Cy TBI (HR 0.63, CI 0.37–1.08; *P*=0.092, borderline significance). Notably, unrelated donors or HLA-Ag mismatching were not identified as risk factors. Meanwhile, a significant association between acute GvHD grade ⩾II and risk of high-level EBV reactivation was also observed (HR 2.51, CI 1.10–5.70; *P*=0.028), although other variables were not predictive for high-level EBV reactivation in this relatively small dataset.

Importantly, a diagnosis of non-Hodgkin lymphoma (NHL) was associated with a highly significant reduction in the risk of EBV reactivation (HR 0.10, CI 0.03–0.33; *P*=0.0001). Thus, of 29 patients with NHL, only 3 developed EBV qPCR positivity (on days 57, 380 and 565). Furthermore, no cases of high-level EBV reactivation were observed for those with NHL in the 2 years after transplant. This was apparent despite equivalent frequency and duration of EBV monitoring in comparison with other patients. Notably, the markedly reduced risk of EBV reactivation observed for patients with NHL was not similarly noted for those with Hodgkin lymphoma; 6/11 (55%) patients with Hodgkin lymphoma exhibited EBV qPCR positivity, including 5 cases of high-level EBV DNAemia.

To establish whether the lack of EBV reactivation observed amongst patients with NHL was independent of other baseline factors, multivariate testing was performed using a Cox proportional hazards model including baseline factors age, diagnosis and transplant conditioning (Table 4). In this analysis, a diagnosis of NHL remained highly predictive for lack of EBV reactivation (HR 0.18, CI 0.05–0.57; *P*=0.004).

### Pre-transplant rituximab

The pre-transplant use of rituximab was examined as a possible determinant of the highly significant reduction in risk of EBV reactivation observed amongst patients with NHL. Rituximab use was documented in 28/29 (97%) patients with NHL, with the last infusion administered a median 3 months (interquartile range 2–5 months) before transplant. Prior rituximab was also recorded in 10/11 (91%) patients with CLL, although the last infusion of rituximab was delivered substantially longer before transplant, at median 15 months (interquartile range 8–26 months). Importantly, only 1/25 (4%) patients who received rituximab within 6 months before transplant reactivated EBV in the first year—a patient with CLL who received rituximab 5 months prior to transplant and who developed high-level EBV reactivation on day 78. In contrast, 7/13 (54%) patients who received rituximab more than 6 months before transplant developed EBV reactivation, including 2 patients with high-level EBV reactivation.

In univariate Cox analysis, there was a highly significant association between pre-transplant rituximab therapy and lack of EBV reactivation, using definitions of pre-transplant rituximab of ‘at any time' prior to transplant (HR 0.34, CI 0.18–0.64; *P*=0.001) or ‘within 6 months' prior to transplant (HR 0.18, CI 0.07–0.48; *P*=0.001). These differences did not persist in multivariate testing, but this analysis was complicated for several reasons. Firstly, there was strong confounding between diagnostic category and prior use of rituximab, such that almost all patients with NHL and CLL received rituximab at some time before transplant. Secondly, it is unclear what constitutes a biologically appropriate cutoff (months before transplant) to define pre-transplant rituximab. Finally, few patients with NHL reactivated EBV, even if they received rituximab more than 6 months before transplant, unlike patients with CLL (Supplementary Table S1).

Regarding the safety of pre-transplant rituximab, testing revealed no significant difference in overall survival for patients treated with rituximab at any time before transplant (HR 0.75, CI 0.42–1.33; *P*=0.317) or within 6 months before transplant (HR 0.76, CI 0.38–1.52; *P*=0.441). Similarly, no significant differences were seen in non-relapse mortality for rituximab given at any time before transplant (HR 1.00 CI 0.46–2.18; *P*=0.994) or using a 6 month cutoff (HR 0.95, CI 0.37–2.43; *P*=0.907). An association between pre-transplant rituximab and risk of either Grade ⩾I or Grade ⩾II acute GvHD was not observed.

## Discussion

To our knowledge, the present study is the largest of its kind to analyse the incidence, kinetics and risk factors for EBV reactivation and PTLD amongst alemtuzumab-treated adult patients undergoing allo-HSCT. We report clinically important rates of these complications, with 48% of individuals exhibiting EBV qPCR positivity by 1 year post transplant and 18% developing high-level EBV reactivation. Furthermore, PTLD was diagnosed in eight patients, giving a crude incidence of 4.3%. This incidence is noticeably greater than that reported in the study of 111 alemtuzumab-treated patients by Carpenter *et al.*,^11^ in which only 0.9% of patients developed PTLD. However, it is apparent that around a third of patients in the latter study received alemtuzumab ‘in the bag' (in which the drug is used to treat the stem cell graft *in vitro* before infusion) rather than *in vivo*; higher peak concentrations and persistence of alemtuzumab have been reported when it is used *in vivo*,^36^ and this is also associated with delayed reconstitution of EBV-specific immunity.^20^ Notably, the incidence of PTLD observed for alemtuzumab-treated patients in the present study remains within the range reported by other smaller series.^6, 7, 8, 9^

Regarding the kinetics of EBV reactivation, most events occurred between 2 and 4 months after transplant, but some cases were documented up to and beyond 12 months. Given this, it is notable that recent guidelines have advised that patients undergoing allo-HSCT at high risk of PTLD should be monitored with EBV qPCR for 3 months post transplant.^33^ The current study supports extending this recommendation to 6 months, at least for alemtuzumab-treated patients. Furthermore, given that greater than 80% of high-level reactivations occurred within 2 weeks of initial EBV qPCR positivity, we suggest that particular scrutiny should be exercised in the first few days following initial EBV qPCR positivity.

Using pre-emptive treatment with rituximab, we observed very good response rates amongst patients who developed high-level EBV reactivation. Thus, 35/38 (92%) of patients exhibited complete resolution of viral DNAemia and disease where present, with no apparent increase in mortality amongst treated patients. Although based on a relatively small number of patients, our findings support the efficacy and safety of rituximab in this setting. However, three patients developed rituximab-refractory PTLD and died from progressive disease despite the use of cytotoxic chemotherapy. Our experience accords with other reports showing poor outcomes with chemotherapy in this setting.^2, 3^ In light of this and other data,^37^ we now aim to treat all patients who develop rituximab-refractory PTLD following allo-HSCT with EBV cytotoxic T lymphocytes or donor-lymphocyte infusion, where practicable.

Analysis of predictors for EBV reactivation revealed that older age at transplant and the occurrence of acute GvHD were significant risk factors. However, our most striking finding was a dramatically reduced incidence of EBV reactivation and PTLD amongst patients with a diagnosis of NHL. These individuals exhibited a highly significant reduction in the risk of EBV reactivation in both univariate and multivariate analyses. None of the 29 patients with NHL exhibited high-level reactivation. Whilst our data should be treated with some caution owing to the retrospective nature of the study, our observation raises the possibility that rituximab delivered prior to transplant might confer a protective effect. In support of this, we found that patients with HL did frequently experience EBV reactivation—HL is not routinely treated with rituximab. Furthermore, our observations are consistent with the anecdotal description by Savani *et al.*^30^ of an absence of EBV reactivation after allo-HSCT in 38 patients who received rituximab prior to, or concurrent with, transplantation. Although similar findings have not been reported in other studies of EBV reactivation and PTLD after allo-HSCT, this may be because these pre-date the routine use of rituximab for patients with NHL^4, 5^ or because they have not specifically analysed the risks associated with NHL.^9, 10, 12, 13, 14, 15, 16^

In light of the above, it is possible that rituximab delivered shortly before (or after) transplant might be a highly effective alternative to pre-emptive strategies for managing PTLD. At the very least, rituximab might be used as prophylaxis for high risk patients, such as those who require anti-thymocyte globulin. Conceivably, prior rituximab might prevent EBV reactivation by delaying post transplant B-cell reconstitution. Indeed, it has a half-life of up to 18 days, with levels remaining detectable in the serum for up to 3 months after administration, and it is known to deplete circulating B cells for around 6 months.^38^ Notably, van Dorp *et al.*^39^ demonstrated a significantly reduced rate of B-cell reconstitution in patients who had received rituximab within 6 months prior to transplant. Alternatively, rituximab might act by reducing the recipient pre-transplant EBV burden. Interestingly, the latter supposes that it is predominantly recipient-derived virus that drives EBV reactivation post allo-HSCT, which might be the case following reduced-intensity conditioning.

To conclude, in this study we have examined EBV reactivation and PTLD amongst a large cohort of adult patients undergoing alemtuzumab TCD allo-HSCT. Importantly, we confirm that alemtuzumab is a clinically important risk factor for the development of PTLD. Furthermore, we observe that the risk of EBV reactivation appears to be greatly reduced amongst patients with NHL who have previously been exposed to rituximab. This suggests that peri-transplant rituximab might be effective prophylaxis for PTLD arising after allo-HSCT. Our data make a strong case for prospectively evaluating the role of rituximab in allograft conditioning.

## Figures and Tables

**Table 1:** Patient characteristics

	*N*	*%*
*Age*		
Median years (range)	51 (17–71)	

*Sex*		
Male	120	65
Female	66	35

*Diagnosis*		
AML/MDS	98	53
NHL	29	16
ALL	18	10
HL	11	6
CLL	11	6
MPD	11	6
Other	8	4

*Donor*		
Unrelated	127	68
Sibling	59	32

*HLA mismatches*		
None	151	81
⩾ 1 Ags	35	19

*Stem cell source*		
PBSC	186	100

*Intensity*		
Reduced intensity	149	80
Myeloablative	37	20

*Conditioning*		
Flu Mel	129	69
Cy TBI	37	20
BEAM +/− Flu	15	8
Other	5	3

*Acute GvHD*		
** **Grade⩾II	66	35

Abbreviations: ALL=acute lymphocytic leukaemia; AML=acute myeloid leukaemia; BEAM=carmustine with etoposide, cytarabine and melphalan; CLL=chronic lymphocytic leukaemia; Cy=cyclophosphamide; Flu=fludarabine; GvHD=graft versus host disease; HLA=human leukocyte antigen; HL=Hodgkin lymphoma; MDS=myelodysplastic syndrome; Mel=melphalan; MPD=myeloproliferative disorder; NHL=non-Hodgkin lymphoma; PBSC=peripheral blood stem cells; TBI=total body irradiation.

**Table 2:** Patients with PTLD after allo-HSCT

*No*.	*Diagnosis*	*Age, years*	*Sex*	*Regimen*	*Donor*	*First EBV DNAemia, day*	*First EBV DNAemia, copies/mL*	*High EBV DNAemia, day*	*High EBV DNAemia, copies/mL*	*Peak EBV DNAemia, copies/mL*	*PTLD diagnosed, day*	*Stage*	*Extranodal disease*	*Histology*^a^	*Outcome, cause of death*	*Follow-up, months*
1	AML	34	M	Cy TBI	MUD	119	71 000	119	71 000	4 346 790	135	IIIB	—	—	Dead, relapse	13
2	ALL	63	M	Flu Mel	Sib	71	193 324	71	193 324	2 704 380	78	IIIB	—	—	Alive	29
3	AML	57	M	Flu Mel	MUD	119	81 686	119	81 686	2 150 650	128	IVB	CNS	DLBCL	Dead, PTLD	5
4	HL	43	M	Flu Mel	MUD	70	5412	84	36 407	57 039	85	IIIA	—	—	Dead, PTLD	8
5	AML	29	F	Cy TBI	MUD	71	33 664	71	33 664	329 542	78	IIIA	—	—	Alive	34
6	MDS	64	M	Flu Mel	MUD	49	712	63	233 774	1 805 490	65	IVB	Bowel	Polymorphic	Dead, PTLD	6
7	AML	57	M	Flu Mel	Sib	84	6405	91	94 945	94 945	92	IIB	—	Polymorphic	Dead, relapse	15
8	ALL	42	F	Flu Mel	MUD	64	7564	70	494 794	494 794	77	IIIA	—	—	Alive	13

Abbreviations: ALL=acute lymphocytic leukaemia; AML=acute myeloid leukaemia; BEAM=carmustine with etoposide, cytarabine and melphalan; CLL=chronic lymphocytic leukaemia; CNS=central nervous system; Cy=cyclophosphamide; DLBCL=diffuse large B-cell lymphoma; Flu=fludarabine; GvHD=graft versus host disease; HLA=human leukocyte antigen; HL=Hodgkin lymphoma; HSCT=haematopoietic stem cell transplantation; MDS=myelodysplastic syndrome; MUD=HLA-matched unrelated donor; Mel=melphalan; MPD=myeloproliferative disorder; NHL=non-Hodgkin lymphoma; PBSC=peripheral blood stem cells; Sib=HLA-matched sibling; TBI=total body irradiation.

a Biopsy material was obtained from three patients—all other patients were diagnosed with probable PTLD.

**Table 3:** Univariate analysis of risk factors for EBV reactivation after allo-HSCT

	*EBV ⩾500 copies/mL*			*EBV ⩾20 000 copies/mL*		
	*HR*	*95% CI*	P *value*	*HR*	*95% CI*	P *value*
*Age*						
⩾50 years	1.54	1.02–2.31	0.039	1.54	0.79–3.01	0.206

*Sex*						
Male versus female	1.22	0.80–1.88	0.360	1.45	0.71–2.99	0.311

*Diagnosis*						
AML/MDS	1.00	—	Ref	1.00	—	Ref
NHL	0.10	0.03–0.33	0.0001	No events	—	—
ALL	0.80	0.41–1.56	0.513	0.67	0.20–2.22	0.510
HL	0.80	0.34–1.84	0.585	1.84	0.70–4.82	0.213
CLL	1.01	0.48–2.11	0.989	0.93	0.28–3.09	0.908
MPD	0.95	0.43–2.10	0.905	0.30	0.04–2.20	0.236
Other	1.26	0.54–2.93	0.591	0.39	0.05–2.89	0.358

*Donor*						
Sibling versus unrelated	1.24	0.83–1.87	0.291	0.64	0.31–1.32	0.226

*HLA mismatches*						
⩾ 1 Ags	0.93	0.55–1.57	0.794	0.87	0.36–2.09	0.760

*Intensity*						
Myeloablative versus RIC	0.76	0.44–1.29	0.309	0.82	0.34–1.97	0.661

*Conditioning*						
Flu Mel	1.00	—	Ref	1.00	—	Ref
Cy TBI	0.63	0.37–1.08	0.092	0.69	0.29–1.65	0.404
BEAM^+^/− Flu	No events	—	—	No events	—	—
Other	0.81	0.25–2.56	0.714	No events	—	—

*Acute GvHD*						
Grade⩾II	1.53	0.91–2.57	0.112	2.51	1.10–5.70	0.028

*Prior rituximab*						
Within 6 months	0.18	0.07–0.48	0.001	0.16	0.02–1.17	0.072
At any time	0.34	0.18–0.64	0.001	0.30	0.09–0.98	0.046

Abbreviations: ALL=acute lymphocytic leukaemia; AML=acute myeloid leukaemia; BEAM=carmustine with etoposide, cytarabine and melphalan; CI=confidence interval; CLL=chronic lymphocytic leukaemia; Cy=cyclophosphamide; Flu=fludarabine; GvHD=graft versus host disease; HLA=human leukocyte antigen; HL=Hodgkin lymphoma; HR=hazard ratio; HSCT=haematopoietic stem cell transplantation; MDS=myelodysplastic syndrome; Mel=melphalan; MPD=myeloproliferative disorder; NHL=non-Hodgkin lymphoma; PBSC=peripheral blood stem cells; Ref=reference category; RIC=reduced-intensity conditioning; TBI=total body irradiation.

**Table 4:** Multivariate analysis of risk factors for EBV reactivation after allo-HSCT

	*EBV ⩾500 copies/mL*		
	*HR*	*95% CI*	P *value*
*Age*			
⩾50 years	1.30	0.76–2.23	0.342

*Diagnosis*			
AML/MDS	1.00	—	Ref
NHL	0.18	0.05–0.57	0.004
ALL	0.89	0.45–1.75	0.734
HL	1.63	0.64–4.16	0.308
CLL	0.87	0.41–1.85	0.724
MPD	0.95	0.43–2.11	0.907
Other	3.01	0.94–9.65	0.063

*Conditioning*			
Flu Mel	1.00	—	Ref
Cy TBI	0.69	0.35–1.36	0.284
BEAM +/− Flu	No events	—	—
Other	0.27	0.05–1.36	0.112

Abbreviations: ALL=acute lymphocytic leukaemia; AML=acute myeloid leukaemia; BEAM=carmustine with etoposide, cytarabine and melphalan; CI=confidence interval; CLL=chronic lymphocytic leukaemia; Cy=cyclophosphamide; Flu=fludarabine; GvHD=graft versus host disease; HLA=human leukocyte antigen; HL=Hodgkin lymphoma; HR=hazard ratio; HSCT=haematopoietic stem cell transplantation; MDS=myelodysplastic syndrome; Mel=melphalan; MPD=myeloproliferative disorder; NHL=non-Hodgkin lymphoma; PBSC=peripheral blood stem cells; Ref=reference category; RIC=reduced-intensity conditioning; TBI=total body irradiation.

## References

[bib1] Heslop HE. How I treat EBV lymphoproliferation. Blood 2009; 114: 4002–4008. 19724053 10.1182/blood-2009-07-143545 PMC2774540

[bib2] Styczynski J, Einsele H, Gil L, Ljungman P. Outcome of treatment of Epstein-Barr virus-related post-transplant lymphoproliferative disorder in hematopoietic stem cell recipients: a comprehensive review of reported cases. Transpl Infect Dis 2009; 11: 383–392. 19558376 10.1111/j.1399-3062.2009.00411.x

[bib3] Fox CP, Burns D, Parker AN, Peggs KS, Harvey CM, Natarajan S et al. EBV-associated post-transplant lymphoproliferative disorder following *in vivo* T-cell-depleted allogeneic transplantation: clinical features, viral load correlates and prognostic factors in the rituximab era. Bone Marrow Transplant 2014; 49: 280–286. 24212561 10.1038/bmt.2013.170

[bib4] Landgren O, Gilbert ES, Rizzo JD, Socie G, Banks PM, Sobocinski KA et al. Risk factors for lymphoproliferative disorders after allogeneic hematopoietic cell transplantation. Blood 2009; 113: 4992–5001. 19264919 10.1182/blood-2008-09-178046 PMC2686146

[bib5] Hale G, Waldmann H. Risks of developing Epstein-Barr virus-related lymphoproliferative disorders after T-cell-depleted marrow transplants. CAMPATH Users. Blood 1998; 91: 3079–3083. 9531622

[bib6] Ho AY, Adams S, Shaikh H, Pagliuca A, Devereux S, Mufti GJ. Fatal donor-derived Epstein-Barr virus-associated post-transplant lymphoproliferative disorder following reduced intensity volunteer-unrelated bone marrow transplant for myelodysplastic syndrome. Bone Marrow Transplant 2002; 29: 867–869. 12058237 10.1038/sj.bmt.1703552

[bib7] Peggs KS, Banerjee L, Thomson K, Mackinnon S. Post transplant lymphoproliferative disorders following reduced intensity conditioning with *in vivo* T cell depletion. Bone Marrow Transplant 2003; 31: 725–726 author reply 727. 12822577 10.1038/sj.bmt.1703893

[bib8] Wagner HJ, Cheng YC, Huls MH, Gee AP, Kuehnle I, Krance RA et al. Prompt versus preemptive intervention for EBV lymphoproliferative disease. Blood 2004; 103: 3979–3981. 14751931 10.1182/blood-2003-12-4287

[bib9] Cohen J, Gandhi M, Naik P, Cubitt D, Rao K, Thaker U et al. Increased incidence of EBV-related disease following paediatric stem cell transplantation with reduced-intensity conditioning. Br J Haematol 2005; 129: 229–239. 15813851 10.1111/j.1365-2141.2005.05439.x

[bib10] Sundin M, Le Blanc K, Ringden O, Barkholt L, Omazic B, Lergin C et al. The role of HLA mismatch, splenectomy and recipient Epstein-Barr virus seronegativity as risk factors in post-transplant lymphoproliferative disorder following allogeneic hematopoietic stem cell transplantation. Haematologica 2006; 91: 1059–1067. 16885046

[bib11] Carpenter B, Haque T, Dimopoulou M, Atkinson C, Roughton M, Grace S et al. Incidence and dynamics of Epstein-Barr virus reactivation after alemtuzumab-based conditioning for allogeneic hematopoietic stem-cell transplantation. Transplantation 2010; 90: 564–570. 20555307 10.1097/TP.0b013e3181e7a3bf

[bib12] Hoegh-Petersen M, Goodyear D, Geddes MN, Liu S, Ugarte-Torres A, Liu Y et al. High incidence of post transplant lymphoproliferative disorder after antithymocyte globulin-based conditioning and ineffective prediction by day 28 EBV-specific T lymphocyte counts. Bone Marrow Transplant 2011; 46: 1104–1112. 21057556 10.1038/bmt.2010.272

[bib13] Bordon V, Padalko E, Benoit Y, Dhooge C, Laureys G. Incidence, kinetics, and risk factors of Epstein-Barr virus viremia in pediatric patients after allogeneic stem cell transplantation. Pediatr Transplant 2012; 16: 144–150. 22288846 10.1111/j.1399-3046.2011.01634.x

[bib14] van der Velden WJ, Mori T, Stevens WB, de Haan AF, Stelma FF, Blijlevens NM et al. Reduced PTLD-related mortality in patients experiencing EBV infection following allo-SCT after the introduction of a protocol incorporating pre-emptive rituximab. Bone Marrow Transplant 2013; 48: 1465–1471. 23749107 10.1038/bmt.2013.84

[bib15] Liu Q, Xuan L, Liu H, Huang F, Zhou H, Fan Z et al. Molecular monitoring and stepwise preemptive therapy for Epstein-Barr virus viremia after allogeneic stem cell transplantation. Am J Hematol 2013; 88: 550–555. 23564232 10.1002/ajh.23452

[bib16] Auger S, Orsini M, Ceballos P, Fegueux N, Kanouni T, Caumes B et al. Controlled Epstein-Barr virus reactivation after allogeneic transplantation is associated with improved survival. Eur J Haematol 2014; 92: 421–428. 24400833 10.1111/ejh.12260

[bib17] Kottaridis PD, Milligan DW, Chopra R, Chakraverty RK, Chakrabarti S, Robinson S et al. *In vivo* CAMPATH-1H prevents graft-versus-host disease following nonmyeloablative stem cell transplantation. Blood 2000; 96: 2419–2425. 11001893

[bib18] Hale G, Cobbold S, Novitzky N, Bunjes D, Willemze R, Prentice HG et al. CAMPATH-1 antibodies in stem-cell transplantation. Cytotherapy 2001; 3: 145–164. 12171722 10.1080/146532401753173981

[bib19] Rebello P, Cwynarski K, Varughese M, Eades A, Apperley JF, Hale G. Pharmacokinetics of CAMPATH-1H in BMT patients. Cytotherapy 2001; 3: 261–267. 12171714 10.1080/146532401317070899

[bib20] Chakrabarti S, Milligan DW, Pillay D, Mackinnon S, Holder K, Kaur N et al. Reconstitution of the Epstein-Barr virus-specific cytotoxic T-lymphocyte response following T-cell-depleted myeloablative and nonmyeloablative allogeneic stem cell transplantation. Blood 2003; 102: 839–842. 12869487 10.1182/blood.V102.3.839

[bib21] Styczynski J, Gil L, Tridello G, Ljungman P, Donnelly JP, van der Velden W et al. Response to rituximab-based therapy and risk factor analysis in Epstein Barr Virus-related lymphoproliferative disorder after hematopoietic stem cell transplant in children and adults: a study from the Infectious Diseases Working Party of the European Group for Blood and Marrow Transplantation. Clin Infect Dis 2013; 57: 794–802. 23771985 10.1093/cid/cit391

[bib22] van Esser JW, Niesters HG, van der Holt B, Meijer E, Osterhaus AD, Gratama JW et al. Prevention of Epstein-Barr virus-lymphoproliferative disease by molecular monitoring and preemptive rituximab in high-risk patients after allogeneic stem cell transplantation. Blood 2002; 99: 4364–4369. 12036863 10.1182/blood.v99.12.4364

[bib23] Comoli P, Basso S, Zecca M, Pagliara D, Baldanti F, Bernardo ME et al. Preemptive therapy of EBV-related lymphoproliferative disease after pediatric haploidentical stem cell transplantation. Am J Transplant 2007; 7: 1648–1655. 17511690 10.1111/j.1600-6143.2007.01823.x

[bib24] Annels NE, Kalpoe JS, Bredius RG, Claas EC, Kroes AC, Hislop AD et al. Management of Epstein-Barr virus (EBV) reactivation after allogeneic stem cell transplantation by simultaneous analysis of EBV DNA load and EBV-specific T cell reconstitution. Clin Infect Dis 2006; 42: 1743–1748. 16705581 10.1086/503838

[bib25] Worth A, Conyers R, Cohen J, Jagani M, Chiesa R, Rao K et al. Pre-emptive rituximab based on viraemia and T cell reconstitution: a highly effective strategy for the prevention of Epstein-Barr virus-associated lymphoproliferative disease following stem cell transplantation. Br J Haematol 2011; 155: 377–385. 21910716 10.1111/j.1365-2141.2011.08855.x

[bib26] Preiksaitis JK, Pang XL, Fox JD, Fenton JM, Caliendo AM, Miller GG. Interlaboratory comparison of epstein-barr virus viral load assays. Am J Transplant 2009; 9: 269–279. 19178414 10.1111/j.1600-6143.2008.02514.x

[bib27] Gil L, Styczynski J, Komarnicki M. Strategy of pre-emptive management of Epstein-Barr virus post-transplant lymphoproliferative disorder after stem cell transplantation: results of European transplant centers survey. Contemp Oncol (Pozn) 2012; 16: 338–340. 23788905 10.5114/wo.2012.30064 PMC3687436

[bib28] Gulley ML, Tang W. Using Epstein-Barr viral load assays to diagnose, monitor, and prevent posttransplant lymphoproliferative disorder. Clin Microbiol Rev 2010; 23: 350–366. 20375356 10.1128/CMR.00006-09 PMC2863367

[bib29] Rooney CM, Smith CA, Ng CY, Loftin SK, Sixbey JW, Gan Y et al. Infusion of cytotoxic T cells for the prevention and treatment of Epstein-Barr virus-induced lymphoma in allogeneic transplant recipients. Blood 1998; 92: 1549–1555. 9716582

[bib30] Savani BN, Pohlmann PR, Jagasia M, Chinratanalab W, Kassim A, Engelhardt B et al. Does peritransplantation use of rituximab reduce the risk of EBV reactivation and PTLPD? Blood 2009; 113: 6263–6264. 19520823 10.1182/blood-2009-04-213892

[bib31] Dominietto A, Tedone E, Soracco M, Bruno B, Raiola AM, Van Lint MT et al. *In vivo* B-cell depletion with rituximab for alternative donor hemopoietic SCT. Bone Marrow Transplant 2012; 47: 101–106. 21460867 10.1038/bmt.2011.28

[bib32] Gallagher A, Armstrong AA, MacKenzie J, Shield L, Khan G, Lake A et al. Detection of Epstein-Barr virus (EBV) genomes in the serum of patients with EBV-associated Hodgkin's disease. Int J Cancer 1999; 84: 442–448. 10404101 10.1002/(sici)1097-0215(19990820)84:4<442::aid-ijc20>3.0.co;2-j

[bib33] Styczynski J, Reusser P, Einsele H, de la Camara R, Cordonnier C, Ward KN et al. Management of HSV, VZV and EBV infections in patients with hematological malignancies and after SCT: guidelines from the Second European Conference on Infections in Leukemia. Bone Marrow Transplant 2009; 43: 757–770. 19043458 10.1038/bmt.2008.386

[bib34] Petropoulou AD, Porcher R, Peffault de Latour R, Xhaard A, Weisdorf D, Ribaud P et al. Increased infection rate after preemptive rituximab treatment for Epstein-Barr virus reactivation after allogeneic hematopoietic stem-cell transplantation. Transplantation 2012; 94: 879–883. 23001354 10.1097/TP.0b013e3182664042

[bib35] McIver Z, Stephens N, Grim A, Barrett AJ. Rituximab administration within 6 months of T cell-depleted allogeneic SCT is associated with prolonged life-threatening cytopenias. Biol Blood Marrow Transplant 2010; 16: 1549–1556. 20580848 10.1016/j.bbmt.2010.05.004 PMC2947610

[bib36] Morris EC, Rebello P, Thomson KJ, Peggs KS, Kyriakou C, Goldstone AH et al. Pharmacokinetics of alemtuzumab used for *in vivo* and *in vitro* T-cell depletion in allogeneic transplantations: relevance for early adoptive immunotherapy and infectious complications. Blood 2003; 102: 404–406. 12623851 10.1182/blood-2002-09-2687

[bib37] Doubrovina E, Oflaz-Sozmen B, Prockop SE, Kernan NA, Abramson S, Teruya-Feldstein J et al. Adoptive immunotherapy with unselected or EBV-specific T cells for biopsy-proven EBV+ lymphomas after allogeneic hematopoietic cell transplantation. Blood 2012; 119: 2644–2656. 22138512 10.1182/blood-2011-08-371971 PMC3311278

[bib38] Pescovitz MD. Rituximab, an anti-cd20 monoclonal antibody: history and mechanism of action. Am J Transplant 2006; 6: 859–866. 16611321 10.1111/j.1600-6143.2006.01288.x

[bib39] van Dorp S, Pietersma F, Wolfl M, Verdonck LF, Petersen EJ, Lokhorst HM et al. Rituximab treatment before reduced-intensity conditioning transplantation associates with a decreased incidence of extensive chronic GVHD. Biol Blood Marrow Transplant 2009; 15: 671–678. 19450751 10.1016/j.bbmt.2009.02.005

