# Supplementary material for: Greatly reduced risk of EBV reactivation in rituximab-experienced recipients of alemtuzumab-conditioned allogeneic HSCT
Source: Bone Marrow Transplant. 2016 Feb 22;51(6):825–32. doi: 10.1038/bmt.2016.19 (PMC4880046; doi:10.1038/bmt.2016.19)
Supplement: Supplementary Figure S1 [file bmt201619x1.docx]

**Figure S1. Patients with Rituximab-Refractory PTLD after Allo-HSCT**

Serial whole blood EBV loads and therapy are depicted for 3 patients with Rituximab-refractory PTLD. The dotted horizontal line indicates the EBV qPCR threshold of sensitivity of 500 copies/ml whole blood; negative results are displayed as 250 copies/ml. R indicates Rituximab infusion; CHOP, cyclophosphamide with doxorubicin, vincristine and prednisolone; and † indicates death.
