# Supplementary material for: Greatly reduced risk of EBV reactivation in rituximab-experienced recipients of alemtuzumab-conditioned allogeneic HSCT
Source: Bone Marrow Transplant. 2016 Feb 22;51(6):825–32. doi: 10.1038/bmt.2016.19 (PMC4880046; doi:10.1038/bmt.2016.19)
Supplement: Supplementary Table S1 [file bmt201619x2.docx]

| **Table S1. Prior Rituximab Therapy and EBV Reactivation after Allo-HSCT** | | | |
| --- | --- | --- | --- |
| Diagnosis | Time of last Rituximab pre-transplant, months | EBV ≥500 copies/ml,  day post-transplant | EBV ≥20 000 copies/ml, day post-transplant |
| NHL | 1 | . | . |
| NHL | 2 | . | . |
| NHL | 2 | . | . |
| NHL | 2 | . | . |
| NHL | 2 | . | . |
| NHL | 2 | . | . |
| NHL | 2 | . | . |
| NHL | 2 | . | . |
| NHL | 2 | 565* | . |
| NHL | 2 | . | . |
| NHL | 2 | . | . |
| NHL | 2 | . | . |
| NHL | 2 | . | . |
| NHL | 2 | . | . |
| CLL | 2 | 372* | . |
| NHL | 3 | . | . |
| NHL | 3 | 380* | . |
| NHL | 4 | . | . |
| NHL | 4 | . | . |
| NHL | 4 | . | . |
| NHL | 4 | . | . |
| NHL | 5 | . | . |
| CLL | 5 | 78 | 78 |
| NHL | 6 | . | . |
| NHL | 6 | . | . |
| NHL | 7 | . | . |
| CLL | 7 | . | . |
| CLL | 10 | 104 | . |
| CLL | 14 | . | . |
| CLL | 15 | 273 | . |
| CLL | 15 | 217 | . |
| NHL | 17 | . | . |
| NHL | 24 | 57 | . |
| NHL | 29 | . | . |
| CLL | 30 | 41 | . |
| NHL | 31 | . | . |
| CLL | 37 | 218 | 337 |
| CLL | 47 | 85 | 99 |
| NHL | None | . | . |
| CLL | None | . | . |
| Abbreviations are explained in Table 1.  * Indicates EBV reactivation events which occurred ≥12 months after transplant. | | | |
